# Antibiotics and RNase P

**DOI:** 10.3390/antibiotics5020015

**Published:** 2016-05-06

**Authors:** Denis Drainas

**Affiliations:** Department of Biological Chemistry, School of Medicine, University of Patras, Rio-Patras 26504, Greece; drainas@med.upatras.gr; Tel.: +30-2610-969127; Fax: +30-2610-969167

**Keywords:** RNase P, neomycin B, neomycin sulfate, puromycin, morpholino oligonucleotides

## Abstract

RNase P is an essential endonuclease in tRNA biogenesis, which generates the mature 5′-termini of tRNAs. Most forms of RNase P are ribonucleoproteins, *i.e.*, they consist of an essential RNA and protein subunits. The catalytic function of ribonucleoprotein RNase P enzymes resides entirely in the RNA subunit. Its high structural and functional diversity among representatives of a vast variety of phylogenetic domains indicates that RNase P could serve as a molecular target and a useful screening system for the development of new drugs in the battle against bacterial drug resistance.

## 1. Introduction

The major problem of modern therapeutics is the ever-increasing frequency of the appearance of resistant bacterial strains to antibiotics, which severely threatens human health. In order to prevent the devastating spreading of infectious diseases, the search for new targets or tools aiming to develop novel antibiotics is of utmost importance. There are accumulated data to support that ribonuclease P (RNase P), the enzyme that is responsible of the cleavage of the 5′ leader sequence of the precursor tRNA, seems to be a promising target or tool in the combat against microbes. RNase P, in most organisms, is a ribonucleoprotein. It consists of an essential RNA subunit and one (bacteria) or more (Archaea and Eukarya) protein subunits [1]. In Bacteria and some Archaea, the RNA subunit is a true *trans*-acting ribozyme, while in Eukarya, it retains residual catalytic activity [2,3,4]. Recently, exceptions that dispute the ribonucleoprotein composition of RNase P have been reported, like the RNase P from human mitochondria [5], the RNase P from *Arabidopsis thaliana* mitochondria, plastids and nucleus [6] and the nuclear RNase P from *Trypanosoma brucei* [7], which do not contain any RNA, and the enzyme activity resides totally in proteins.

The ribonucleoprotein character of RNase P with fundamental differences in structure and function between bacterial and eukaryotic RNase P enzymes makes the bacterial enzyme an ideal molecular model for the study of various significant inhibitors. In the past two decades, RNase P was extensively studied as a drug target, using either antisense strategies or classical inhibitors [8]. This review focuses on drugs that target bacterial RNase P, and we will give special reference to a new class of inhibitors that act as antibiotics, the peptide–morpholino oligonucleotide conjugates, designed to bind and promote the cleavage of specific mRNAs by the action of RNase P as tools inhibiting gene function and bacterial growth.

## 2. Ribosome-Targeting Antibiotics

Antibiotics developed so far are based on natural products, such as aminoglycosides, macrolides and several peptidyl transferase inhibitors, among others, predominantly targeting 30S and 50S ribosomal subunits and blocking protein synthesis in specific pathogens, but not in mammalian cells. The detailed structural data on ribosome provides a useful tool for the design of specific inhibitors, even for each pathogen individually [9]. The ubiquitous and essential endonuclease RNase P, due to its ribonucleoprotein character, could be characterized as a small ribosome. RNase P seems to be capable of serving as both a molecular target for a variety of drugs and a reliable screening system for their biological activity. The structural differences between bacterial and eukaryotic RNase P enzymes make RNase P an ideal *in vitro* molecular model for the study of various important ribosome-targeting inhibitors.

**Figure 1:**
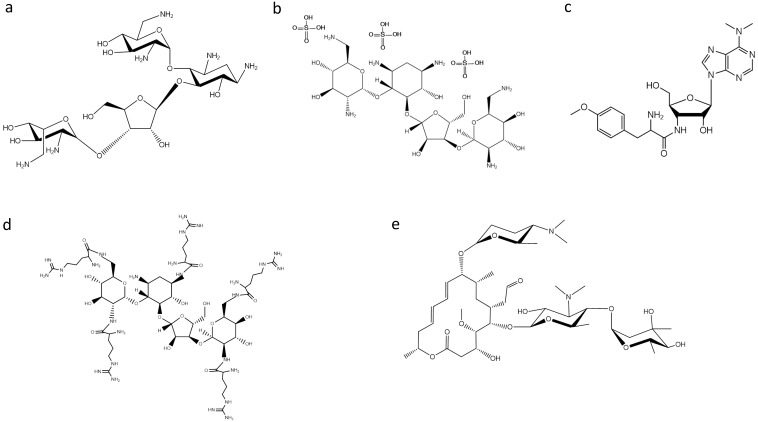
Structures of: (**a**) neomycin B; (**b**) neomycin sulfate; (**c**) puromycin; (**d**) aminoglycoside derivative NeoR5; and (**e**) spiramycin.

Bacterial RNase P was found to be inhibited by puromycin and several aminoglycoside antibiotics [10,11]. One of the most studied classes of antibiotics on RNase P activity are aminoglycosides. Aminoglycosides, such as neomycin B (NeoB) (Figure 1), tobramycin, paromomycin, gentamycin and kanamycin, are positively-charged compounds that can interact with various RNA molecules displaying a wide array of effects. They act by displaying essential Mg^2+^ ions by protonated amino groups, and the fact that the inhibition is pH dependent indicates a direct relationship between the available positively-charged amino groups in these antibiotics and their inhibitory potency [11]. Based on this observation, Gopalan and coworkers made an effort to improve the inhibitory potency of aminoglycosides on bacterial RNase P by conjugation of arginine, guanidinium and lysyl residues [12,13]. They found that neomycin B penta-arginyl conjugate (NeoR5) is the most potent inhibitor among aminoglycosides having a considerably small IC_50_ value (0.5 μM). Additionally, under identical assay conditions, both hexa-lysyl derivative (NeoK6) and hexa-guanidium derivative (NeoG6) of neomycin B were 10-fold weaker, while NeoB was 800-fold weaker than NeoR5. Moreover, it is interesting to note that NeoR5 showed a different effectiveness on type A and type M archaeal RNase P RNA; the catalytically-active RNase P RNA (type A) was significantly inhibited, while the inactive RNase P RNA (type M) was modestly activated [13]. It was concluded that the inhibitor’s potency depends on the molecular backbone, as well as the length, flexibility and composition of the side chains. Furthermore, in a recent study, neomycin sulfate (Figure 1) was found to potentiate the antimicrobial properties of mucroporin toward *Staphylococcus aureus*, an antimicrobial agent that inhibits bacterial isoleucyl-tRNA synthetase-mediated Ile-tRNA aminoacylation and protein translation [14]. Dunman and colleagues [15] screened a drug library approved by the Food and Drug Administration. Among the compounds tested, the aminoglycoside antibiotic neomycin sulfate, which is approved for topical use, exhibited the most potent activity against the tested strain in the absence and presence of mupirocin, suggesting that the ability of neomycin sulfate to potentiate mupirocin may be mediated partially by its ability to inhibit RNase P activity. Moreover, topical application of the combination displayed significantly improved murine nasal decolonization toward a panel of *S. aureus* strains, in comparison to either agent when tested alone. Likewise, the combination led to the near elimination of methicillin-resistant and high-level mupirocin-resistant strains in a murine wound model of colonization.

Apart from aminoglycosides, peptidyl transferase inhibitors, like puromycin, (Figure 1), have been found to inhibit RNase P activity. Puromycin, a mimic of the 3′ terminal end of the aminoacyl-tRNA, was the first inhibitor of RNase P activity reported [10]. Furthermore, it is interesting to make a special reference to the peculiar behavior of macrolides on bacterial RNase P. Drainas and colleagues reported that macrolides, such as spiramycin (Figure 1), erythromycin, tylosin and roxithromycin, affected the *Escherichia* *coli* holoenzyme and RNase P RNA (M1 RNA)-alone reaction in a low micromolar range, where they acted as dose-dependent activators [16]. Detailed analysis of the activation by spiramycin revealed a mixed-type activation mode with an 18-fold increase of kcat/Ks in the holoenzyme reaction and a 12-fold one in the RNA-alone reaction. Ribozyme activators are rarely described, but one could notice that there have been no significant efforts toward this direction.

## 3. Searching for New Inhibitors

The need for more potent inhibitors capable of overcoming the drug resistance that has been developed by bacterial pathogens has led several groups to screen libraries of compounds aiming to isolate small molecule inhibitors with the strong inhibitory effect of RNase P activity that can be used as chemical scaffolds to develop new drugs.

**Figure 2:**
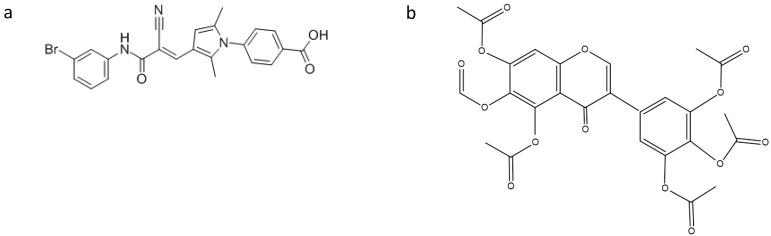
Structures of: (**a**) RNPA 1000; and (**b**) iriginol hexaacetate.

Olson and colleagues [17] reported that the protein subunit of RNase P (RnpA) from *Staphylococcus aureus* plays a role in mRNA degradation. This finding, in combination with the fact that RnpA is an essential *S. aureus* enzyme, led the authors to consider RnpA as a suitable target for antimicrobial drug discovery. Indeed, by using a fluorescence-based high throughput assay, they identified small molecules that inhibit RnpA’s RNA degradation activity *in vitro*. One of these compounds, namely RNPA 1000 (Figure 2), was shown to inhibit *S. aureus* cellular mRNA turnover. Moreover, it exhibited antimicrobial activity against methicillin-resistant (MRSA), vancomycin intermediate (VISA) and vancomycin-resistant (VRSA) *S. aureus*, as well as other pathogenic bacteria, like *Staphylococcus epidermidis*, *Streptococcus pneumoniae*, *Streptococcus pyogenes*, *Streptococcus agalactiae* and *Bacillus cereus*, with high RnpA conservation, and limited bacterial lethal effect in a murine acute lethal model of infection [17]. These results indicate that the essential protein subunit of the ribonuclease RNase P is an attractive target for antimicrobial drug development.

Recently, Chen and Fierke [18] reported the development of a real-time fluorescence polarization/anisotropy (FP/FA) assay for measuring RNase P activity using a 5′ end fluorescein-labeled pre-tRNA^Asp^ as a substrate. Using this assay, they carried out a high-throughput screen of a library of 2880 compounds. From these compounds, only one, namely iriginol hexaacetate (Ir6Ac) (Figure 2), was confirmed as an inhibitor for *Bacillus subtilis* RNase P. Detailed kinetic analysis revealed that Ir6Ac inhibits RNase P following a mixed type of inhibition with a considerably small K_i_ value (130 nM), indicating that this compound has high affinity for RNase P.

These high throughput methods give one the ability to screen thousands of compounds in order to identify new small molecule inhibitors that act as a bacteriostatic or a bactericidal. Although they seem to be promising to overcome the problem of drug resistance, the innate capability of bacteria to develop resistance at a rate that exceeds the development of new drugs suggests that the existing methods for the development of viable and long-term anti-microbial treatments are doomed to failure. Without alternative strategies, the development of drug resistance by pathogenic bacteria appears as one of the most significant threats of human health in the 21st century. Towards this line, Altman and colleagues, in an effort to overcome this problem, applied a new strategy. To kill bacteria, they used morpholino oligonucleotides (MOs) as external guide sequences (EGS) that are complementary to bacterial RNA targets, covalently linked with a cell-penetrating peptide (CPP). EGS are designed in such way to form a structure resembling a portion of the natural tRNA substrates when it hybridizes to the target RNA [19]. This leads to specific cleavage of the target RNA by RNase P, thereby inactivating the pathogenic RNA. More specifically, they designed a peptide-morpholino oligomer conjugate (PMO) that targets a highly conserved region in the gyrase A gene of *E. coli* that is effective at inactivating gyrase A in many bacteria [20]. Furthermore, in another study, they applied this strategy to an infected mouse cutaneous wound model [21]. They designed a PMO conjugate that targets *S. aureus* gyrase A mRNA, reducing the bacterial growth by site-specific mRNA cleavage via RNase P. Wounds treated with a single dose of PMO conjugate displayed improved healing that was associated with increased epithelialization, reduced bacterial load and increased matrix deposition. The transition from drug sensitivity to drug resistance is attributed to the fact that currently-used antibiotics are small molecules that are susceptible to one mutation. The use of nucleic acids as part of a novel compound could remove the problem of resistance development. PMO seems to have the advantage of not being susceptible to resistance due to genetic mutations and is more effective against drug-resistant strains.

## 4. Conclusions

RNase P represents an essential enzyme that has changed our view of molecular evolution and has become the first biochemical evidence for the existence of the “RNA world”. Today, almost 40 years after its discovery, it still intrigues, as it holds an important position in the transition to the RNP world. RNase P, due to its ribonucleoprotein character, resembles the ribosome and can be used for similar studies toward the understanding of the drugs’ mode of action and, more importantly, for the design of drugs specifically targeting RNase P. All of the above-mentioned data clearly show that several compounds apart from their well-known suppressive action on protein synthesis are also capable of directly affecting tRNA biogenesis by inhibiting RNase P activity through mechanisms that, however, require further investigation. RNase P represents a reliable biological target and a screening system for many compounds that might be helpful in the selection and clinical application of new and more potent agents and in the better understanding of the mode of their therapeutic action. Additionally, the development of peptide-morpholino oligonucleotide conjugates as novel inhibitors that are not easily susceptible to bacterial resistance, designed to attack pathogenic RNAs and be cleaved by cell’s RNase P, highlights RNase P not only as a target, but also as a tool in the battle against pathogenic bacteria.

## References

[1] Hartmann E., Hartmann R.K. (2003). The enigma of ribonuclease P evolution. Trends Genet..

[2] Guerrier-Takada C., Gardiner K., Marsh T., Pace N., Altman S. (1983). The RNA moiety of ribonuclease P is the catalytic subunit of the enzyme. Cell.

[3] Pannucci J.A., Haas E.S., Hall T.A., Harris J.K., Brown J.W. (1999). RNase P RNAs from some Archaea are catalytically active. Proc. Natl. Acad. Sci. USA.

[4] Kikovska E., Svard S.G., Kirsebom L.A. (2007). Eukaryotic RNase P RNA mediates cleavage in the absence of protein. Proc. Natl. Acad. Sci. USA.

[5] Holzmann J., Frank P., Löffler E., Bennett K.L., Gerner C., Rossmanith W. (2008). RNase P without RNA: Identification and functional reconstitution of the human mitochondrial tRNA processing enzyme. Cell.

[6] Gutmann B., Gobert A., Giegé P. (2012). PRORP proteins support RNase P activity in both organelles and the nucleus in *Arabidopsis*. Genes Dev..

[7] Taschner A., Weber C., Buzet A., Hartmann R.K., Hartig A., Rossmanith W. (2012). Nuclear RNase P of Trypanosoma brucei: A single protein in place of the multicomponent RNA-protein complex. Cell Rep..

[8] Toumpeki C., Stamatopoulou V., Bikou M., Grafanaki K., Kallia-Raftopoulou S., Papaioannou D., Stathopoulos C., Drainas D., Gualerzi C.O., Brandi L., Fabbretti A., Pon C.L. (2013). Targeting Ribonuclease P. Antibiotics: Targets, Mechanisms and Resistance.

[9] Steitz T.A., Moore P.B. (2003). RNA, the first macromolecular catalyst: The ribosome is a ribozyme. Trends Biochem. Sci..

[10] Vioque A. (1989). Protein synthesis inhibitors and catalytic RNA. Effect of puromycin on tRNA precursor processing by the RNA component of Escherichia coli RNase P. FEBS Lett..

[11] Mikkelsen N.E., Brannvall M., Virtanen A., Kirsebom L.A. (1999). Inhibition of RNase P RNA cleavage by aminoglycosides. Proc. Natl. Acad. Sci. USA.

[12] Eubank T.D., Biswas R., Jovanovic M., Litovchick A., Lapidot A., Gopalan V. (2002). Inhibition of bacterial RNase P by aminoglycoside-arginine conjugates. FEBS Lett..

[13] Kawamoto S.A., Sudhahar C.G., Hatfield C.L., Sun J., Behrman E.J., Gopalan V. (2008). Studies on the mechanism of inhibition of bacterial ribonuclease P by aminoglycoside derivatives. Nucleic Acids Res..

[14] Hughes J., Mellows G. (1980). Interaction of pseudomonic acid A with Escherichia coli B isoleucyl-tRNA synthetase. Biochem. J..

[15] Blanchard C., Brooks L., Beckley A., Colquhoun J., Dewhurst S., Dunman P.M. (2015). Neomycin Sulfate Improves the Antimicrobial Activity of Mupirocin based Antibacterial Ointments. Antimicrob. Agents Chemother..

[16] Toumpeki C., Vourekas A., Kalavrizioti D., Stamatopoulou V., Drainas D. (2008). Activation of bacterial ribonuclease P by macrolides. Biochemistry.

[17] Olson P.D., Kuechenmeister L.J., Anderson K.L., Daily S., Beenken K.E., Roux C.M., Reniere M.L., Lewis T., Weiss W.J., Pulse M. (2011). Small molecule inhibitors of Staphylococcus aureus RnpA alter cellular mRNA turnover, exhibit antimicrobial activity, and attenuate pathogenesis. PLoS Pathog..

[18] Liu X., Chen Y., Fierke C.A. (2014). A real-time fluorescence polarization activity assay to screen for inhibitors of bacterial ribonuclease P. Nucleic Acids Res..

[19] Gopalan V., Vioque A., Altman S. (2002). RNase P: Variations and uses. J. Biol. Chem..

[20] Wesolowski D., Tae H.S., Gandotra N., Llopis P., Shen N., Altman S. (2011). Basic peptide-morpholino oligomer conjugate that is very effective in killing bacteria by gene-specific and nonspecific modes. Proc. Natl. Acad. Sci. USA.

[21] Sawyer A.J., Wesolowski D., Gandotra N., Stojadinovic A., Izadjoo M., Altman S., Kyriakides T.R. (2013). A peptide-morpholino oligomer conjugate targeting Staphylococcus aureus gyrA mRNA improves healing in an infected mouse cutaneous wound model. Int. J. Pharm..

